# A Reaction‐Induced Localization of Spin Density Enables Thermal C−H Bond Activation of Methane by Pristine FeC_4_^+^

**DOI:** 10.1002/chem.201902572

**Published:** 2019-08-13

**Authors:** Caiyun Geng, Jilai Li, Thomas Weiske, Helmut Schwarz

**Affiliations:** ^1^ Institute of Theoretical Chemistry Jilin University 130023 Changchun P. R. China; ^2^ Institut für Chemie Technische Universität Berlin Straße des 17. Juni 115 10623 Berlin Germany

**Keywords:** gas-phase reaction, hydrogen-atom transfer, metal carbide, methane activation, quantum chemical calculation

## Abstract

The reactivity of the cationic metal‐carbon cluster FeC_4_^+^ towards methane has been studied experimentally using Fourier‐transform ion cyclotron resonance mass spectrometry and computationally by high‐level quantum chemical calculations. At room temperature, FeC_4_H^+^ is formed as the main ionic product, and the experimental findings are substantiated by labeling experiments. According to extensive quantum chemical calculations, the C−H bond activation step proceeds through a radical‐based hydrogen‐atom transfer (HAT) mechanism. This finding is quite unexpected because the initial spin density at the terminal carbon atom of FeC_4_^+^, which serves as the hydrogen acceptor site, is low. However, in the course of forming an encounter complex, an electron from the doubly occupied *sp*‐orbital of the terminal carbon atom of FeC_4_^+^ migrates to the singly occupied π*‐orbital; the latter is delocalized over the entire carbon chain. Thus, a highly localized spin density is generated *in situ* at the terminal carbon atom. Consequently, homolytic C−H bond activation occurs without the obligation to pay a considerable energy penalty that is usually required for HAT involving closed‐shell acceptor sites. The mechanistic insights provided by this combined experimental/computational study extend the understanding of methane activation by transition‐metal carbides and add a new facet to the dizzying mechanistic landscape of hydrogen‐atom transfer.

## Graphical abstract

**Getting together**: Radical‐type hydrogen‐atom transfer transpires by localized spin density generated *in situ* once the reaction partners approach each other.

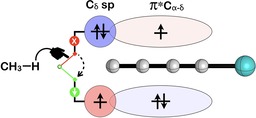

## Introduction

Transfer of a hydrogen atom constitutes a key step in a broad range of chemical, environmental, and biological processes.^1^ In this context, particular attention has been paid to the activation and valorization of methane. For the first step of the C−H bond activation of this rather inert hydrocarbon, various mechanistic scenarios were identified depending on the given conditions, such as hydrogen‐atom transfer (HAT),^2^ proton‐ coupled electron transfer (PCET),2b, 3 and hydride transfer (HT).2b, 4

Previous investigations, in which metal oxides were often used as prototypical model systems, suggested that the presence of a pronounced and spatially confined high spin density at the reaction‐initiating center constitutes a prerequisite for an efficient, radical‐based HAT process.2a, 2b, 2d, 2e, 5 If the spin density at the H‐abstractor site decreases, the energy barrier for HAT increases.2d, 2e, 5b, 6 The mechanism of a homolytic bond cleavage may then switch to either a PCET wherein a Lewis acid‐base [M^δ+^−O^δ‐^] unit serves as an active site,2b, 3 or to a HT depending on the electrophilicity of the active center in, for example, diatomic MC^+^ (M=Cu, and Au).2b, 4 Furthermore, in a recent systematic investigation of the C−H bond activation of methane by the whole series of the diatomic 3d transition‐metal carbide cations MC^+^ (M=Sc‐Zn),^7^ quite a rich mechanistic landscape was identified, covering all three scenarios mentioned above.^7^

As to the role of the spin density in HAT, Ye and Neese demonstrated that HAT from ethane to an active site with depleted spin density is associated with a distinct energy barrier; this is due to the absence of a “prepared” acceptor site of the metal‐oxo group.^8^ Similarly, Shaik and co‐workers showed that HAT to a closed‐shell molecule in general requires a higher barrier owing to the additional promotion energy needed to create a high spin density at the active center.5b Further examples can be found in refs. 2d, 5a, 9. These and other observations2d, 5, 6 raise the general question as to whether a prepared state must indeed be present at the active site to initiate efficient HAT, or if a significant high‐spin density can be generated along the reaction coordinate without paying the penalty of a high promotion energy.1h, 2a, 2b, 2d, 2e, 5, 10

Herein, we report an entirely unexpected finding on the reaction of the pristine FeC_4_^+^ cluster with methane as revealed in a combined experimental/computational approach. The gas‐phase experiments were performed by using Fourier‐transform ion cyclotron resonance mass spectrometry (FT‐ICR MS) under thermal, single‐collision conditions. Mechanistic aspects were elucidated by high‐level quantum mechanical calculations, which enabled a detailed analysis of the changes of the electronic structure along the reaction coordinate. As will be shown, this study adds another facet to the rich landscape of HAT mechanisms by demonstrating that, in the course of a reaction, a poorly reactive site with a low spin density can be transformed into a highly reactive one carrying high spin density. The knowledge gained from this exercise may open up a new perspective for methane activation through the use of carbon‐based materials in heterogeneous catalysis.2a, 11

## Results and Discussion

### Experimental results

**Figure 1:**
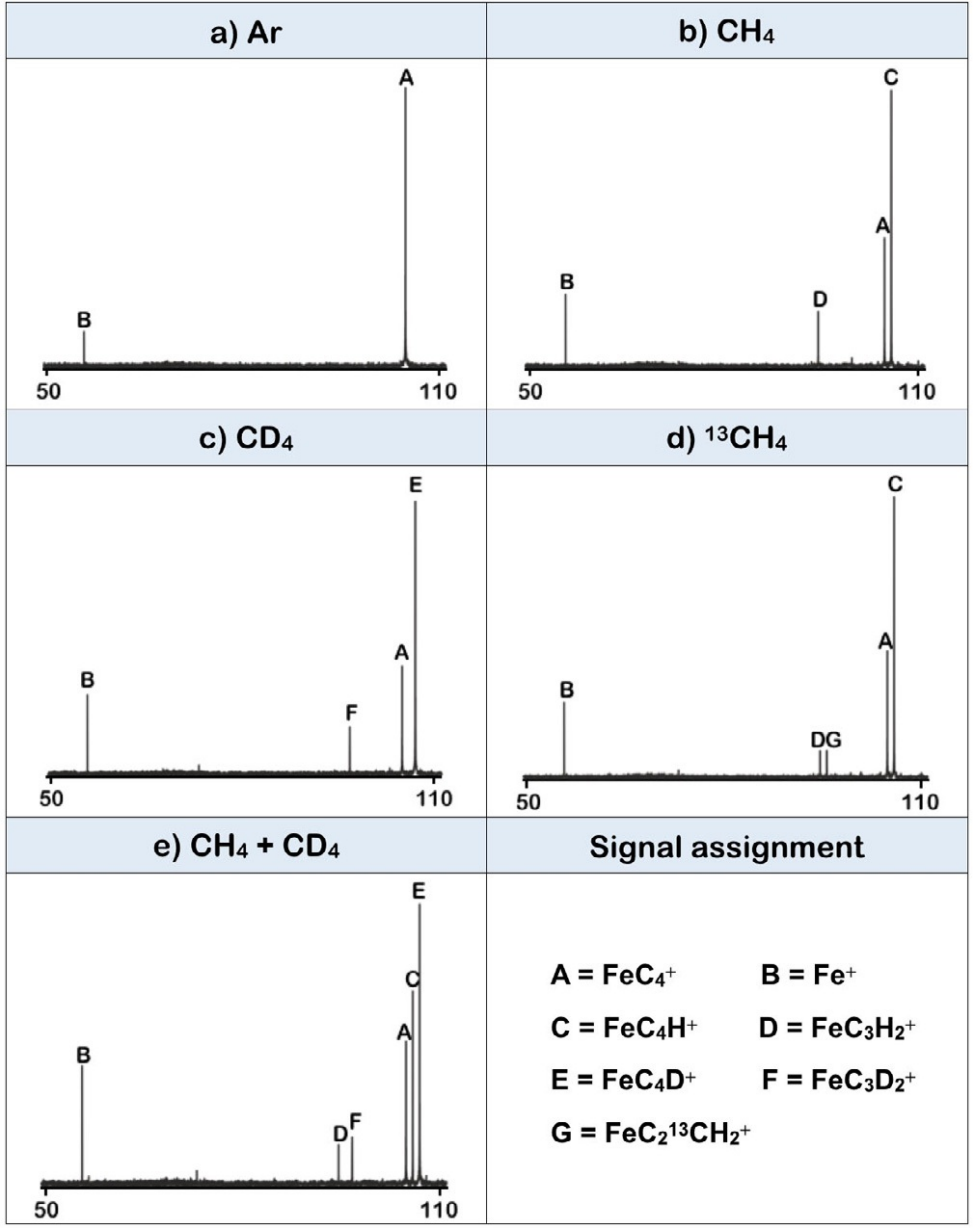
Mass spectra for the thermal reaction of FeC_4_^+^ with a) Ar at 2.0×10^−8^ mbar, b) CH_4_ at 2.0×10^−8^ mbar, c) CD_4_ at 3.0×10^−8^ mbar, d) ^13^CH_4_ at 2.0×10^−8^ mbar, and e) a 1:2 mixture of CH_4_ and CD_4_ at 3.0×10^−8^ mbar after a reaction time of 3s, respectively. All *x*‐axes are scaled in *m*/*z*, and the *y*‐axes are normalized relative ion abundances.

The reactions were conducted by using FT‐ICR‐MS (for details, see the Experimental Section in the Supporting Information). The FT‐ICR mass spectra, Figure 1, show the reactions of mass‐selected, thermalized FeC_4_^+^ ions (*m*/*z=*104) with CH_4_, CD_4_, ^13^CH_4_, and a 1:2 mixture of CH_4_ and CD_4_ (see refs. 3b, 6b for details). To differentiate between reactions of the parent ion with background gases, a reference spectrum with argon has been recorded as well (Figure 1 a).

As shown in the reference spectrum (Figure 1 a), when only argon is admitted to the ICR cell, a signal **B** with Δ*m*=−48 relative to the precursor ion FeC_4_^+^ appears; this signal is assigned to the product ion Fe^+^, indicating that FeC_4_^+^ reacts by carbon‐atom transfer with background gases. Upon leaking CH_4_ into the ICR cell at a stationary pressure of 2.0×10^−8^ mbar, a new signal **C** with Δ*m*=+1 relative to FeC_4_^+^ is identified as FeC_4_H^+^; clearly, hydrogen‐atom transfer from methane to the precursor ion is accompanied by the release of CH_3_^.^ (Equation 1 a 
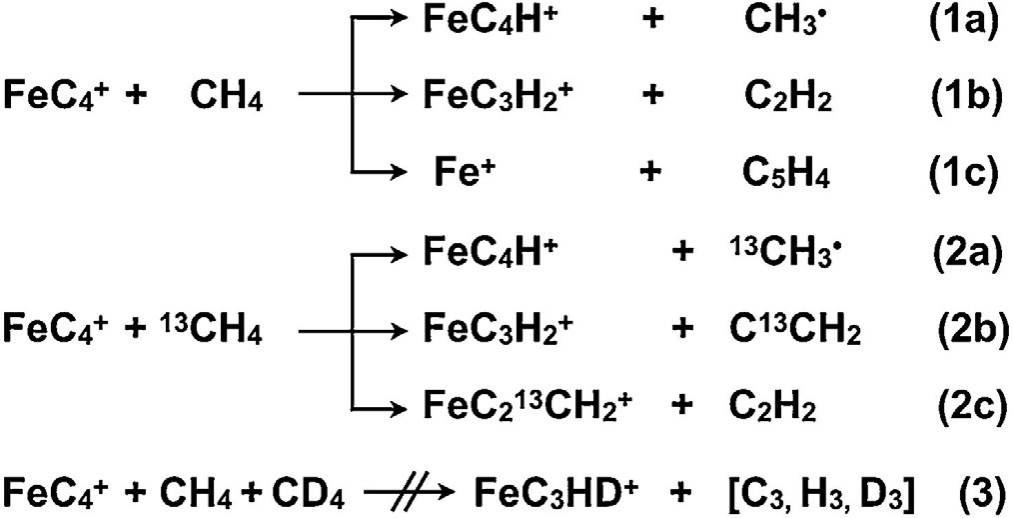
, Figure 1 b). In addition, a signal **D** with Δ*m*=−10 appears. This signal is identified as FeC_3_H_2_^+^ and is formed by the formal transfer of two hydrogen atoms from methane to FeC_4_^+^ accompanied by the back‐transfer of a carbon atom and its coupling with CH_2_ to eventually release acetylene, C_2_H_2_ (Equation 1 b, Figure 1 b). As seen from a comparison of Figures 1 a, b, the contribution of Equation 1 c to the generation of the reaction products seems negligible.

By using CD_4_, signals **E** (FeC_4_D^+^) and **F** (FeC_3_D_2_^+^) appear (Figure 1 c). If FeC_4_^+^ is exposed to ^13^CH_4_, in addition to **C** (FeC_4_H^+^) and **D** (FeC_3_H_2_^+^) in Figure 1 b, a new signal, **G** (FeC_2_^13^CH_2_^+^), is identified, (Equations 2 a–c, Figure 1 d). Clearly, a carbon‐atom exchange in the FeC_4_^+^/^13^CH_4_ couple precedes the C−C coupling and the generation of acetylene. However, while the ratio of the signals **D** and **G** precludes a complete equilibration of the whole carbon‐atom pool, an extensive exchange process must have taken place. Finally, a 1:2 mixture of CH_4_ and CD_4_ was introduced into the ICR cell to exclude the possibility of multiple reactive collisions to be responsible for the formation of FeC_3_H_2_^+^. This is confirmed by the absence of a signal for FeC_3_HD^+^ (Equation 3).

Clearly, the experimental findings reveal that FeC_4_^+^ activates methane at ambient temperature. The rate constant k(FeC_4_^+^/CH_4_) is estimated to 5.7×10^−10^ cm^3^ molecule^−1^ s^−1^. This corresponds to a collision efficiency of *ϕ*=53 %.^12^ The intermolecular kinetic isotope effect (KIE) derived from the FeC_4_^+^/CH_4_/CD_4_ couples, corrected for background contributions, amounts to 1.4. In addition to the labelling experiments, the elementary compositions of the charged species have been confirmed by exact mass measurements.

### Quantum chemical (QC) calculations

#### The most stable structure and molecular properties of FeC_4_^+^

According to our quite elaborate calculations, performed at the NEVPT2(17e,15o)/QZ//CASSCF(17e,15o)/TZ level of theory, the structure of the most stable isomer of FeC_4_^+^ consists of a linear arrangement of the five atoms having an iron atom at one end of the rod. Other isomers like the spoke and hub (Fe^+^) arrangement or an Fe^+^ atom decorated by two C_2_ ligands can be ruled out; they are much higher in energy (for details, see Table 1 and Figure S1). The linear FeC_4_^+^ exists in two nearly isoenergetic electronic states: one corresponds to a sextet state (^6^[FeC_4_]^+^) and the other to a quartet state (^4^[FeC_4_]^+^).^13^ Due to the small energy difference (<3 kJ mol^−1^), which is within the error bar of the applied QC method, we are reluctant to assign which of the two forms the ground state; moreover, at room temperature FeC_4_^+^ may exist as a mixture of the two states.

**Table 1:** Bond distances in Angstroms [Å], bond angles in degrees [°], charges in |e|, and spin densities in *μ*_B_ of FeC_4_^+^ obtained at the NEVPT2(17e,15o)/QZ//CASSCF(17e,15o)/TZ level of theory.

	^6^[FeC_4_]^+^	^4^[FeC_4_]^+^
	Bond Length	
*r*_Cδ‐Cγ_	1.293	1.294
*r*_Cγ‐Cβ_	1.321	1.331
*r*_Cβ‐Cα_	1.246	1.242
*r*_Cα‐Fe_	1.913	1.935
	
	Bond Angle	
∢C_β_C_γ_C_δ_	180	180
	
	Charge	
C_δ_	0.13	0.12
C_γ_	−0.08	−0.07
C_β_	−0.08	−0.09
C_α_	−0.11	−0.11
Fe	1.15	1.14
	
	Spin Density	
C_δ_	0.35	−0.16
C_γ_	0.35	−0.21
C_β_	0.10	0.00
C_α_	0.26	−0.11
Fe	3.94	3.48

As to the possible effects of the spin states on various molecular features of FeC_4_^+^, the following was noted: the individual bond length distances of FeC_4_^+^ are similar, irrespective of the electronic state. The same holds true for the atomic charge distributions. For both states of FeC_4_^+^ almost all of the positive charge is located at the terminal iron atom; C_δ_ carries less than 0.13|e|, whereas minor negative charges are built up at the remaining three carbon atoms C_α_, C_β_, and C_γ_. Only the distribution of the spin density exhibits some differences. However, for either spin state, most of the unpaired electrons are located at the iron atom; except for C_β_ all carbon atoms of the carbide chain carry some spin density but never more than 0.35 *μ*_B_.

#### The potential energy surface describing the interaction of FeC_4_^+^ with CH_4_

To obtain mechanistic insight into the FeC_4_^+^‐mediated C−H bond activation of methane, QC calculations based on the density functional theory (DFT)^14^ method were carried out; for details, see the Computational Section in the Supporting Information. As shown earlier,2b, 4a, 7, 15 the decisive step in the thermal activation of CH_4_ almost always corresponds to the cleavage of the first C−H bond of the substrate and not so much to the subsequent coupling reactions. In addition, our extensive DFT‐based calculations demonstrate the extreme complexity of the follow‐up processes. Therefore, the present study primarily addresses four representative pathways for the initial phase of methane activation by FeC_4_^+^; for the sake of clarity, details of all other remaining pathways, considered in this study, are transferred to the Supporting Information (Figures S2–S3).

**Figure 2:**
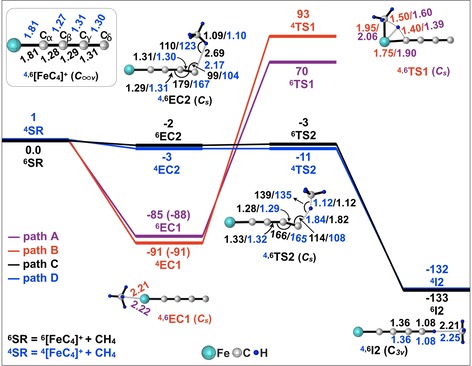
Simplified potential energy surfaces (Δ*H*_298K_ in kJ mol^−1^) obtained at the ωB97XD/QZ//ωB97/TZ level of theory for the C−H bond cleavage steps in the reaction of FeC_4_^+^ with CH_4_. Key structures with selected geometric parameters are also provided. Bond lengths are given in Angstroms [Å] and angles in degrees [°].

Although neutral and possessing only a negligibly permanent electric dipole moment,^16^ methane approaches the rod‐shaped ^4, 6^[FeC_4_]^+^ preferentially from the side of the terminal iron atom. This reflects the fact that most of the positive charge is concentrated at the metal (see Table 1). As displayed in Figure 2, for the two spin states in the reaction with CH_4_ the nearly linear encounter complexes ^4,6^EC1 are formed by strong electrostatic interaction through the reaction paths A (purple) and B (red). Although ^4,6^EC1 correspond to the energetically preferred arrangements, these very intermediates prove to be a dead end in the further course of a hydrogen‐atom transfer to C_α_ of the FeC_4_ moiety. Both transition states ^6^TS1 and ^4^TS1 to bring about HAT are with 70 and 93 kJ mol^−1^, respectively, located well above the entrance asymptote and without external energy supply not accessible. It was noted in passing that the geometries of the transition states ^4,6^TS1 are surprisingly similar to those of the FeC^+^/CH_4_ system.^7^ The energetically hot ^4,6^EC1 only have the choices either to be stabilized by collisional cooling or by the emission of IR‐photons; however, these processes are quite inefficient under the present conditions. Thus, ^4,6^EC1 can only revert to the reactants.

On the other hand, a van der Waals interaction of methane with the terminal carbon‐atom (C_δ_) of FeC_4_^+^, in a side‐on fashion, leads to the formation of the rather flexible and loosely bound encounter complexes ^6^EC2 and ^4^EC2 through paths C (black) and D (blue), respectively. In both cases, the intermediates ^4,6^EC2 are converted barrier‐free to ^4,6^I2 through ^4,6^TS2. As ^4, 6^TS2 are located below the entrance asymptote, FeC_4_^+^ is able to activate methane at ambient temperature. In any case, the driving force to generate the rather stable insertion species ^4, 6^I2 is mostly due to a favorable thermochemistry: The energetic requirements in weakening the C_β_−C_γ_ bond of the carbon chain as indicated by the change of the bond lengths from 1.29 to 1.36 Å and the cleavage of the C−H bond of methane are overcompensated by the formation of a rather strong C−H bond with an *sp*‐hybridized carbon atom at the end of the carbon chain.^17^

Finally, detachment of the loosely bound CH_3_‐radical from the intermediates ^6^I2 and ^4^I2 leads to the main product couple FeC_4_H^+^/CH_3_^.^. As to the minor product shown in Figures 1 b, c, that is, loss of C_2_H_2_, this reaction may proceed through the rather complex ‘rebound‐isomerization’ channels as displayed in Figure S3. As this reaction does not form the focus of the present work, details of the process will not be discussed here.

#### A closer inspection of the mechanism of the C−H bond cleavage

**Figure 3:**
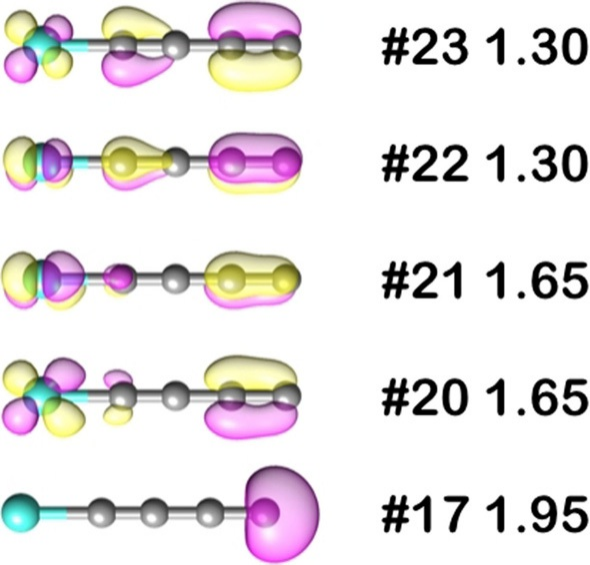
Schematic orbital diagrams for ^6^[FeC_4_]^+^ obtained at the CASSCF(17e,15o)/TZ level of theory. Natural orbital partial occupation numbers are given. See Figure S4 for the complete collection of orbitals.

In the following, the process proceeding on the sextet state is discussed in detail. For the sake of clarity, the discussion of the quartet state is moved to the SI. Figure 3 shows the schematic frontier orbital diagrams of ^6^[FeC_4_]^+^ obtained at the CASSCF level of theory.

Three reaction mechanisms were considered for the hydrogen‐atom transfer:

i) *Proton coupled electron transfer*: As displayed in Figure 3, the doubly occupied *sp*‐orbital at C_δ_ may serve as a potential proton acceptor. To maximize the overlap of orbitals, the optimal angle of C_δ_‐H‐C_CH4_ should be about 180°. Since deprotonation of methane has a standard reaction enthalpy of about 1745 kJ mol^−1^, even the formation of a strong C−H bond to an *sp*‐hybridized carbon atom cannot compensate the energy required for the *heterolytic* cleavage of the C−H bond in methane; rather, the PCET process must be endothermic as the calculated reaction enthalpy to produce FeC_3_CH^2+^/CH_3_^−^ amounts to over 1200 kJ mol^−1^. Furthermore, the bond order data (<0.12) for ^6^TS2 indicate that the interaction between the CH_3_ moiety and FeC_4_ is negligible. Thus, for thermodynamic reasons, the reaction through a PCET channel does not appear to be feasible.

ii) *Hydride transfer*: A closer look at the frontier orbitals of ^6^[FeC_4_]^+^ reveals that all suitable π‐orbitals of the carbon atoms to accept an electron pair, which are sufficiently low in energy, are already doubly or at least singly occupied. Therefore, ^6^[FeC_4_]^+^ does not meet the prerequisites to activate methane through a HT channel.^7^

iii) *Hydrogen‐atom transfer*: Numerous studies have demonstrated that a significant spin density at the hydrogen‐acceptor site plays a crucial role in the thermal, single‐collision activation of methane.2d, 2e, 5, 18 If the spin is delocalized, as for instance in [Mg_2_O_2_]^⋅+^,6b the apparent barriers towards H‐abstraction from CH_4_ are located well above the entrance channel. As listed in Table 1, the C_δ_ atom owns a low spin density of only 0.35 *μ*_B_. The spin density is not only locally depleted, it also obstructs the HAT process further by being distributed over two different π*‐orbitals.

At a first glance, it seems that all of the established mechanisms cannot explain what happens in the course of hydrogen transfer from methane to FeC_4_^+^ (Equation 1 a).

**Figure 4:**
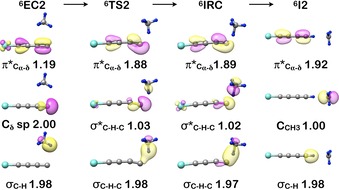
Schematic orbital diagrams represented by a frontier orbital analysis for the selected points in path C of Figure 2 as obtained by CASSCF(19e,17o) calculations. Natural orbital partial occupation numbers are also given.

Figure 4 displays the schematic frontier orbital diagrams of the C−H bond activation step. For the sextet state along the reaction coordinate, the H‐transfer undoubtedly follows a HAT mechanism; this is supported by the fact that, as required by theory,3b, 5b in the transition state there is a node at the hydrogen atom in transit at the three‐center/three‐electron bond (3c/3e); the latter involves the atoms C_δ_, H_T_, and C${}_{\mathrm{CH}{}_{4}}$ . This 3c/3e bond is comprised by a doubly occupied σ_C‐H‐C_‐orbital and a singly occupied σ*_C‐H‐C_‐orbital.

**Figure 5:**
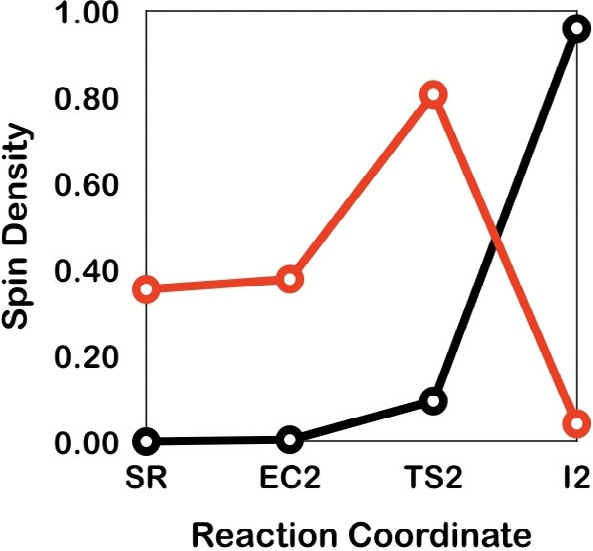
The evolution of the spin density at C_δ_ (red) and C_CH4_ (black) along the reaction coordinate of the C−H bond activation of methane by ^6^[FeC_4_]^+^.

Next, Figure 5 shows the evolution of the spin density at C_δ_ and C${}_{\mathrm{CH}{}_{4}}$ for the stationary points during the C−H bond activation process. It clearly demonstrates that when the two reactants encounter, the approaching methane molecule induces a significant increase in spin density at C_δ_, especially at the transit from the encounter complex ^6^EC2 to the transition state ^6^TS2. When ^6^TS2 is converted to the intermediate ^6^I2, the electron of the moving H‐atom pairs with the single electron at the C_δ_‐atom to finally form a σ_C‐H_‐bond. As a consequence, the spin‐density at C_δ_ almost completely disappears and shifts to the ensuing carbon atom of the CH_3_‐fragment (Figure 5). This finding convincingly reinforces the conclusion already drawn that the sextet state of FeC_4_^+^ takes over a hydrogen atom from methane by the classical HAT mechanism. Although the corresponding spin densities in the quartet state are not as high as in the sextet, the spin density at the reactive center C_δ_ also increases upon the approach of the methane molecule, thereby facilitating the H‐transfer through a radical mechanism (Figures S5 and S6).

Why can the reaction proceed through a radical pathway under thermal, single‐collision conditions even though the initial spin density is low at the reactive site?

As proposed by Shaik5b and Neese,^8^ to form a reactive metal‐oxyl radical from a metal‐oxo intermediate, a highly energy‐demanding preparatory step involving decoupling and elongating of a M=O bond is required. For example, the energy necessary for the elongation of the Fe−O_oxo_ bond in the quintet oxo‐iron(IV) intermediate from its equilibrium geometry to the transition state amounts to about 39 kJ mol^−1^.^8^

A closer inspection of the structures shown in Figure 2 reveals that in going from the separated reactants to the transition state structure, even though it requires an energetically unfavorable elongation of the C_β_−C_γ_ bond and some bending of the C_β_‐C_γ_‐C_δ_ moiety, the associated contraction of the C_δ_−C_γ_ and C_α_−C_β_ bonds efficiently compensates the energy requirement for the “preparatory step” (see Figure 2). As expected, for ^6^[FeC_4_]^+^, the deformation energy of the FeC_4_ moiety in going from the separated reactants to the transition state amounts to only 12 kJ mol^−1^ (obtained at the ωB97XD level of theory). In addition, as proposed by Nørskov,2c the hydrogen affinity of a catalyst plays a key role in the radical C−H bond activation. Thus, the favorable thermochemistry in the C−H bond activation step as shown in Figure 2 also pulls down the potential energy surface according to Hammond's postulate.^19^

Finally, the crucial question centers on how methane induces the localization of spin density at the terminal C_δ_ of FeC_4_^+^?

If methane were to interact only with the singly occupied π*‐orbital, which is delocalized over the entire carbon chain, a similar reactivity of the individual carbon atoms of the rod should be expected. In addition, the optimum angle C_δ_‐H‐C${}_{\mathrm{CH}{}_{4}}$ should be close to 90°. But this is not the case. In fact, the respective transition states are much higher for the reactions involving the other carbon sites (Figure S2). Furthermore, the angle C_δ_‐H‐C${}_{\mathrm{CH}{}_{4}}$ in ^6^TS2 is with 114° much larger than the optimum value.

A closer inspection of the frontier orbital diagram shown in Figure 4 reveals that the electron pair occupying the *sp*‐orbital at C_δ_ is not innocent. The localization of spin density is ascribed to the electronic reorganization between the doubly occupied *sp*‐orbital at C_δ_ and the singly occupied delocalized π*‐orbital along the carbon chain. When methane approaches the terminal C_δ_‐atom, a slight bending occurs at the terminal carbon chain (<C_β_‐C_γ_‐C_*δ*_=166°). This eases the migration of one of the electrons of the doubly occupied *sp*‐orbital at C_δ_ to the delocalized π*‐orbital. As a result, a radical site with high spin density is generated *in situ* at the terminal carbon atom C_δ_, which eventually leads to the radical‐like transition state ^6^TS2.

## Conclusions

Novel and unprecedented mechanistic insights into the FeC_4_^+^‐mediated activation of methane have been obtained by means of Fourier‐transform ion cyclotron resonance mass spectrometry in combination with high‐level quantum chemical calculations. As shown experimentally, FeC_4_^+^ activates the C−H bond of methane at ambient temperature. The reaction is very efficient and proceeds by a radical‐based, classical hydrogen‐atom transfer. This mechanistic scenario is quite surprising as the initial spin density is depleted at the terminal carbon atom. The root causes why nevertheless HAT occurs were addressed by a detailed frontier orbital analysis. As a complement to Neese's and Shaik's hypothesis on the electronic requirements of metal‐oxo intermediates in C−H bond activation, the present study demonstrates on how an active radical center can be generated *in situ* along the reaction coordinate without a significant energy penalty to pay. The mechanistic insights gained from this study may aid the rational design of carbon‐based catalysts for C−H bond activation.

## Conflict of interest

The authors declare no conflict of interest.

## Supporting information

As a service to our authors and readers, this journal provides supporting information supplied by the authors. Such materials are peer reviewed and may be re‐organized for online delivery, but are not copy‐edited or typeset. Technical support issues arising from supporting information (other than missing files) should be addressed to the authors.

Supplementary
