# Supplementary material for: A Reaction‐Induced Localization of Spin Density Enables Thermal C−H Bond Activation of Methane by Pristine FeC4 +
Source: Chemistry. 2019 Aug 13;25(56):12940–5. doi: 10.1002/chem.201902572 (PMC6852486; doi:10.1002/chem.201902572)
Supplement: Supplementary file 1 — Supplementary [file CHEM-25-12940-s001.pdf]

CHEMISTRY
A European Journal

Supporting Information

A Reaction-Induced Localization of Spin Density Enables Thermal C—H Bond Activation of Methane by Pristine FeC_4^+

Caiyun Geng,[b] Jilai Li,*[a, b] Thomas Weiske,[b] and Helmut Schwarz*[b]

chem_201902572_sm_miscellaneous_information.pdf

Table of Contents

1. Experimental Details 2
2. Computational Details 2
3. Figures 4
4. Tables 12
5. References 14
6. Coordinates 16

1. Experimental Details

The ion/molecule reactions were performed with a Spectrospin CMS 47X Fourier transform ion cyclotron resonance (FT-ICR) mass spectrometer equipped with an external ion source as described elsewhere.[1-3] In brief, FeC_4^+ was generated by laser ablation of a compressed iron/graphite powder (1:4; molar ratio) disk using a Nd:YAG laser operating at 532 nm; helium served as a cooling and carrier gas. Using a series of potentials and ion lenses, the ions were transferred into the ICR cell, which is positioned in the bore of a 7.05 T superconducting magnet. After thermalization by pulses of argon (ca. 2×10^{-6} mbar), the reactions of mass-selected FeC_4^+ were studied by introducing isotopologues of methane, i.e. CH_4, CD_4, $^{13}CH_4$, and a mixture of CH_4 and CD_4 (CH_4 : CD_4 = 1:2) via leak valves at stationary pressures. A temperature of 298 K was assumed for the thermalized clusters.[1-3]

2. Computational Details

The calculations of the electronic structures were performed with Gaussian 16 and ORCA.[4,5]

To locate the most stable structure of FeC_4^+, a Fortran-based genetic algorithm[6] to generate initial guess structures of FeC_4^+, followed by density functional theory (DFT) calculations, were conducted; these results point to **A01** as the most stable species (Figure S1), in agreement with a previous study.[7] The most stable structure of FeC_4^+ corresponds to a linear arrangement of these five atoms with the iron atom located at one end of the carbon chain.

To elucidate whether the ground state of **A01** corresponds to a sextet or a quartet state, quite elaborate multireference (MR) calculations were conducted. The state-specific complete active space self-consistent field (CASSCF)[8] approach in conjunction with def2-TZVP (TZ) basis set,[9,10] as implemented in ORCA 4, was employed to optimize the geometries of **A01**. An active space (17e,15o) is considered in these MR calculations; for selection of the active space, see Figure S4. Finally, *n*-electron valence perturbation theory (NEVPT2)[11] single-point energy (SPE) calculations were performed by using the def2-QZVP (QZ) basis set.[9,10]

We used the ωB97 density functional in combination with the TZ basis set for structural optimization to model the potential energy surfaces of the reaction.[12,13] Harmonic vibrational frequencies were computed to verify the nature of the stationary points. The minimum structures reported in this paper show only positive eigenvalues of the Hessian matrix, whereas the transition states (TSs) have only one negative eigenvalue. Intrinsic reaction coordinate (IRC)[14-17] calculations were also performed to confirm that the transition states correlate between designated intermediates. The thermodynamic functions (ΔH) were estimated within the ideal gas, rigid-rotor, and harmonic oscillator approximations at 298 K and 1 atm.

We also conducted further calculations for structural optimization by using the BH&HLYP,[18] MN15-L,[19] MN15,[20] ωB97XD[12] functionals combined with TZ basis set for ^{4,6}TS1 and ^{4,6}TS2; these structures are shown in Tables S1 and S2.

As commonly accepted, the geometries of molecular structures are less dependent on the level of theory than their energies. For further energetic refinements, SPE calculations at the ωB97XD/QZ level of theory were performed on the structures optimized with ωB97 functional.

As suggested by Perdew and co-workers,[21] the electron density distributions and atomic charges were calculated at the CASSCF(19e,17o)/TZ level of theory to avoid physically meaningless electron density obtained by DFT method.

3. Figures

Figure S1. Most stable isomers of FeC_4^+ as calculated at the ωB97/TZ level of theory. Selected geometric parameters are also provided. Bond lengths are given in Å. The superscripts refer to the respective spin states. The symmetries and relative enthalpies (kJ mol^{-1}) are given in parentheses. Color codes: sextet state, black; quartet state, blue.

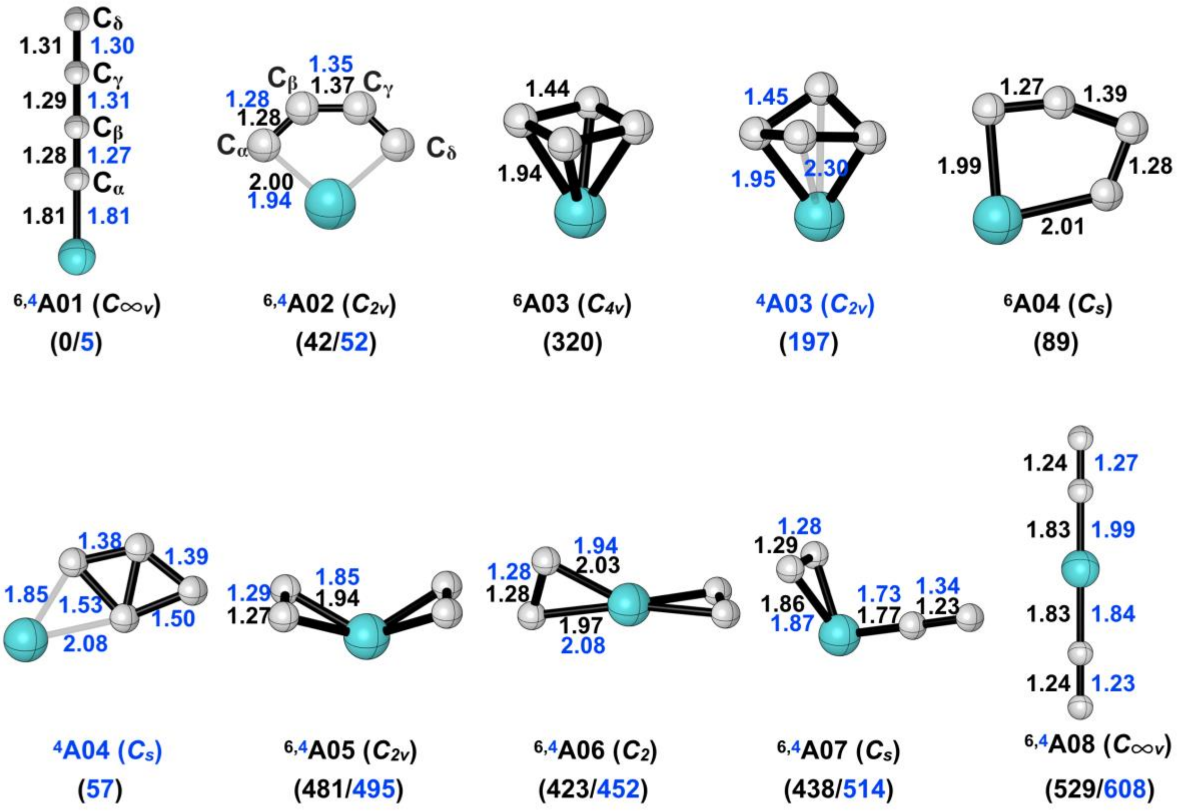

Figure S2. Transition states related to active sites of C_β and C_γ. Relative energies (ΔH in kJ mol^{-1} are also provided in parentheses. Selected geometric parameters are also provided. Charges are omitted for the sake of clarity. Bond lengths are given in Å and angles in degrees. The relative enthalpies (kJ mol^{-1}) are given in parentheses. Color codes: sextet state, black; quartet state, blue.

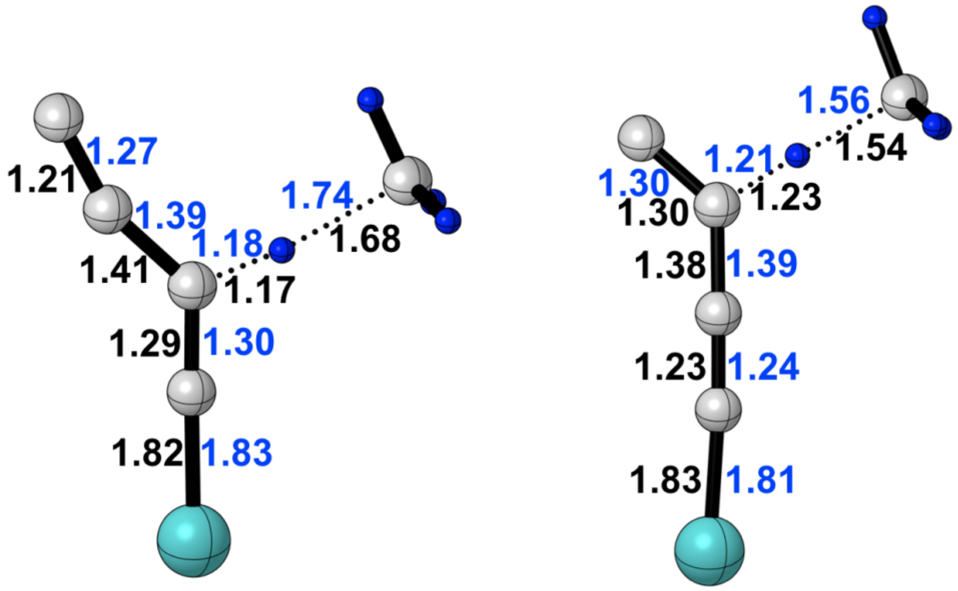

^{6,4}TSC$_\beta$ (168/185)

^{6,4}TSC$_\gamma$ (66/62)

Figure S3. Simplified potential energy profiles calculated at the ωB97XD/QZ//ωB97/TZ level of theory with geometric structures (a, b and c; ΔH_{298K} in kJ mol^{-1}) for the reactions of FeC_4^+ with CH_4. Charges are omitted for the sake of clarity.

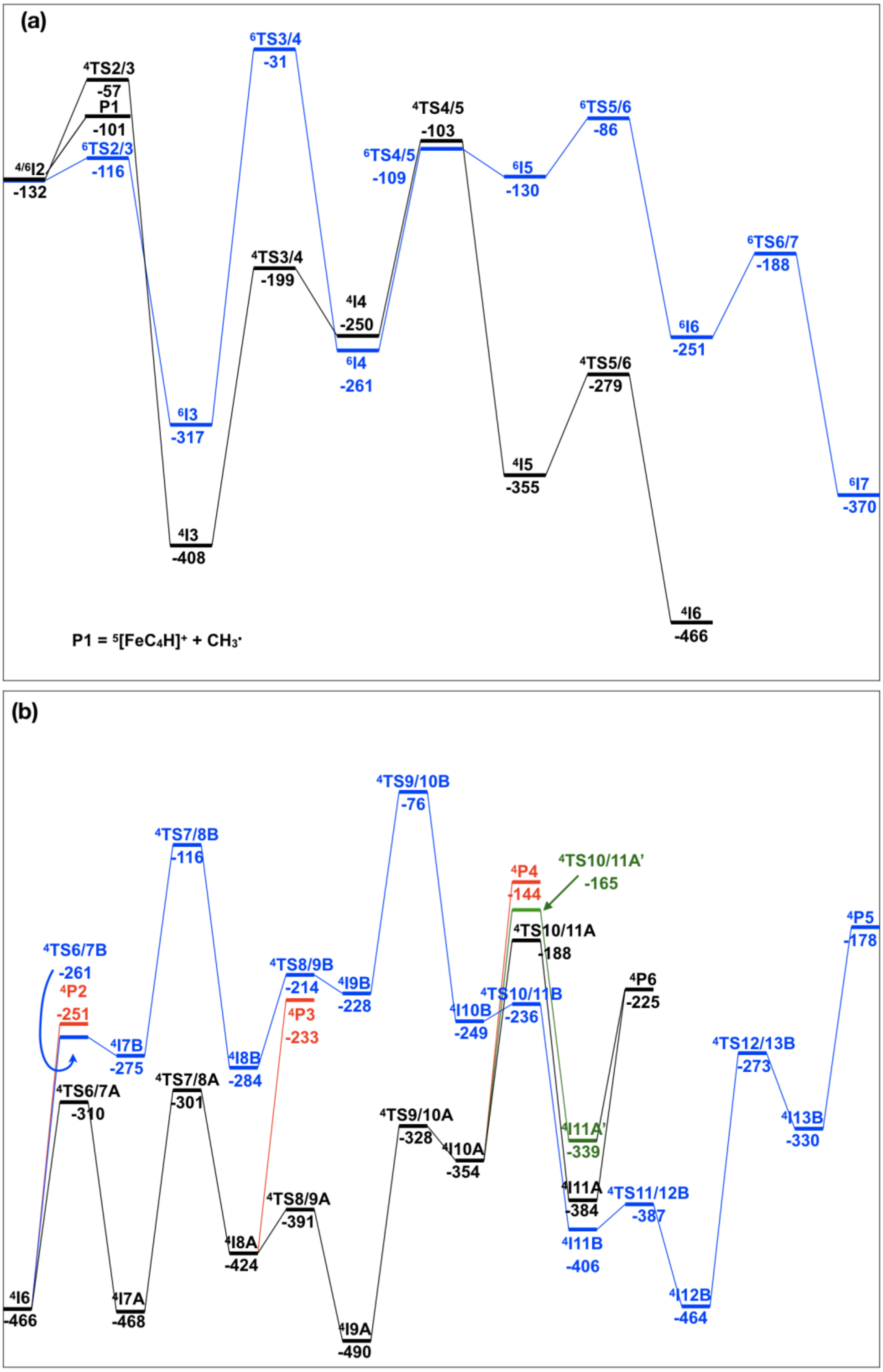

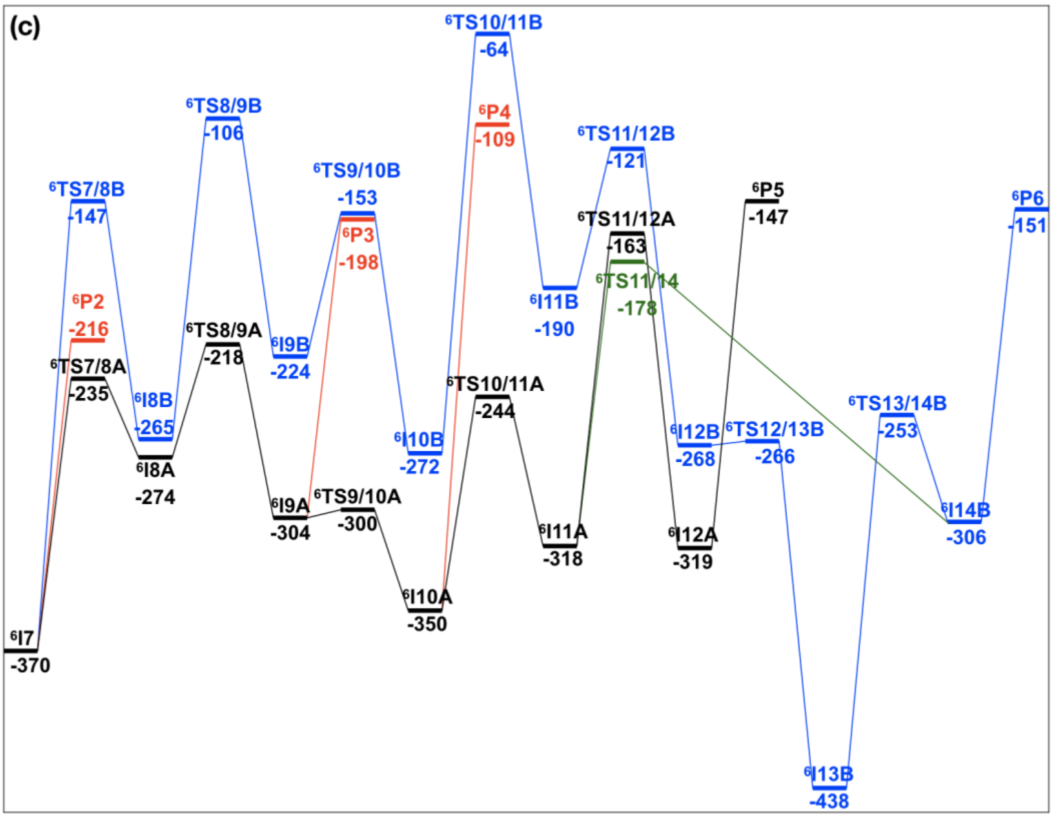

(c)
⁶I7
-370
⁶TS7/8B
-147
⁶P2
-216
⁶TS7/8A
-235
⁶I8B
-265
⁶I8A
-274
⁶TS8/9B
-106
⁶TS8/9A
-218
⁶I9B
-224
⁶I9A
-304
⁶TS9/10B
-153
⁶P3
-198
⁶TS9/10A
-300
⁶I10B
-272
⁶I10A
-350
⁶TS10/11B
-64
⁶P4
-109
⁶TS10/11A
-244
⁶I11B
-190
⁶I11A
-318
⁶TS11/12B
-121
⁶TS11/12A
-163
⁶TS11/14
-178
⁶I12B
-268
⁶I12A
-319
⁶P5
-147
⁶TS12/13B
-266
⁶I13B
-438
⁶TS13/14B
-253
⁶I14B
-306
⁶P6
-151

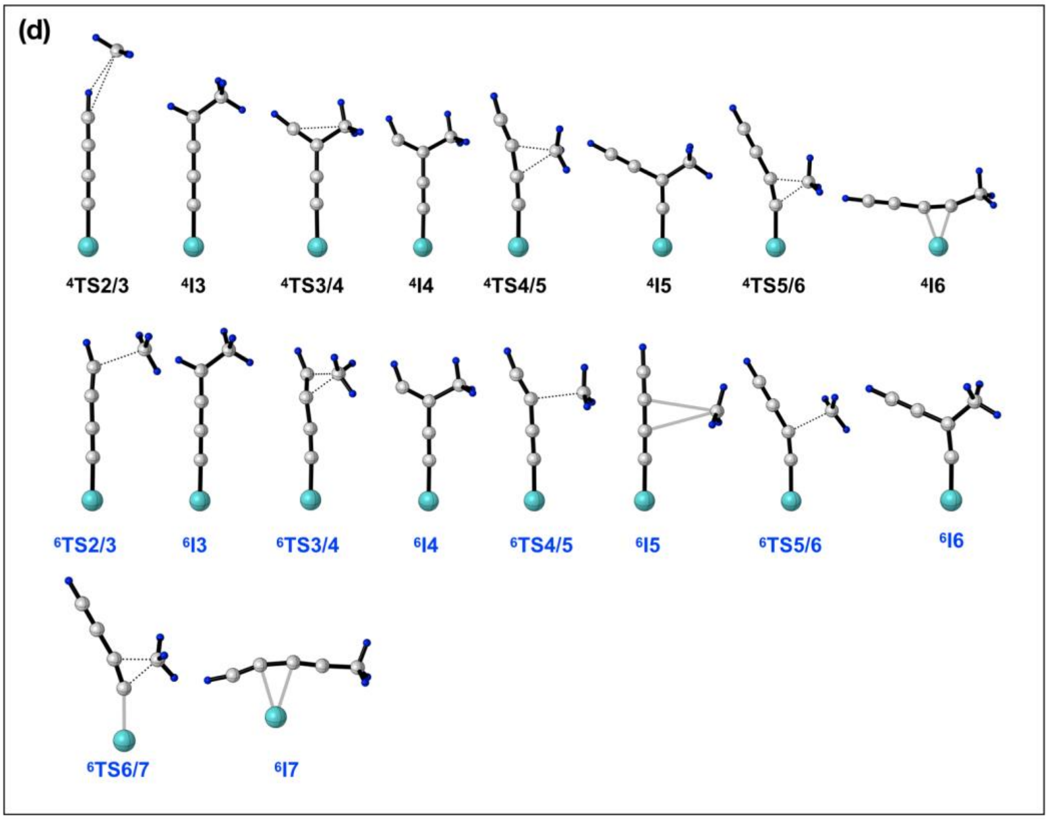

(d)
⁴TS2/3
⁴I3
⁴TS3/4
⁴I4
⁴TS4/5
⁴I5
⁴TS5/6
⁴I6
⁶TS2/3
⁶I3
⁶TS3/4
⁶I4
⁶TS4/5
⁶I5
⁶TS5/6
⁶I6
⁶TS6/7
⁶I7

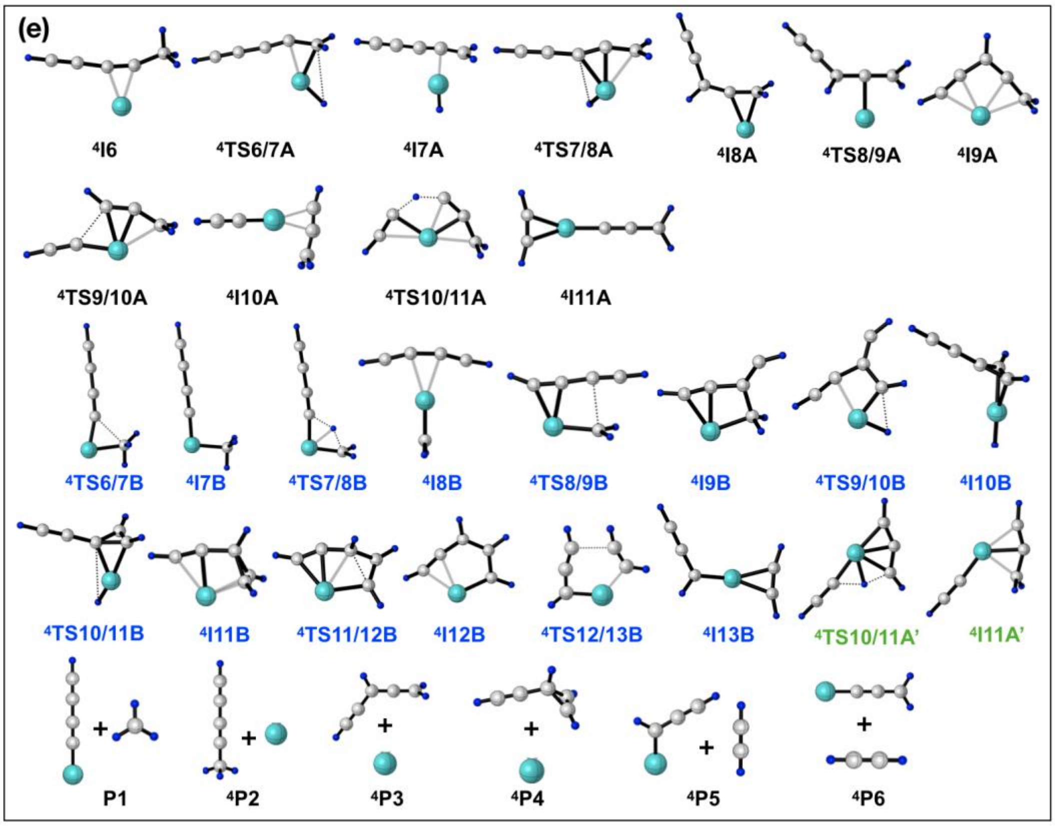
(e)
⁴I6
⁴TS6/7A
⁴I7A
⁴TS7/8A
⁴I8A
⁴TS8/9A
⁴I9A
⁴TS9/10A
⁴I10A
⁴TS10/11A
⁴I11A
⁴TS6/7B
⁴I7B
⁴TS7/8B
⁴I8B
⁴TS8/9B
⁴I9B
⁴TS9/10B
⁴I10B
⁴TS10/11B
⁴I11B
⁴TS11/12B
⁴I12B
⁴TS12/13B
⁴I13B
⁴TS10/11A'
⁴I11A'
P1
⁴P2
⁴P3
⁴P4
⁴P5
⁴P6

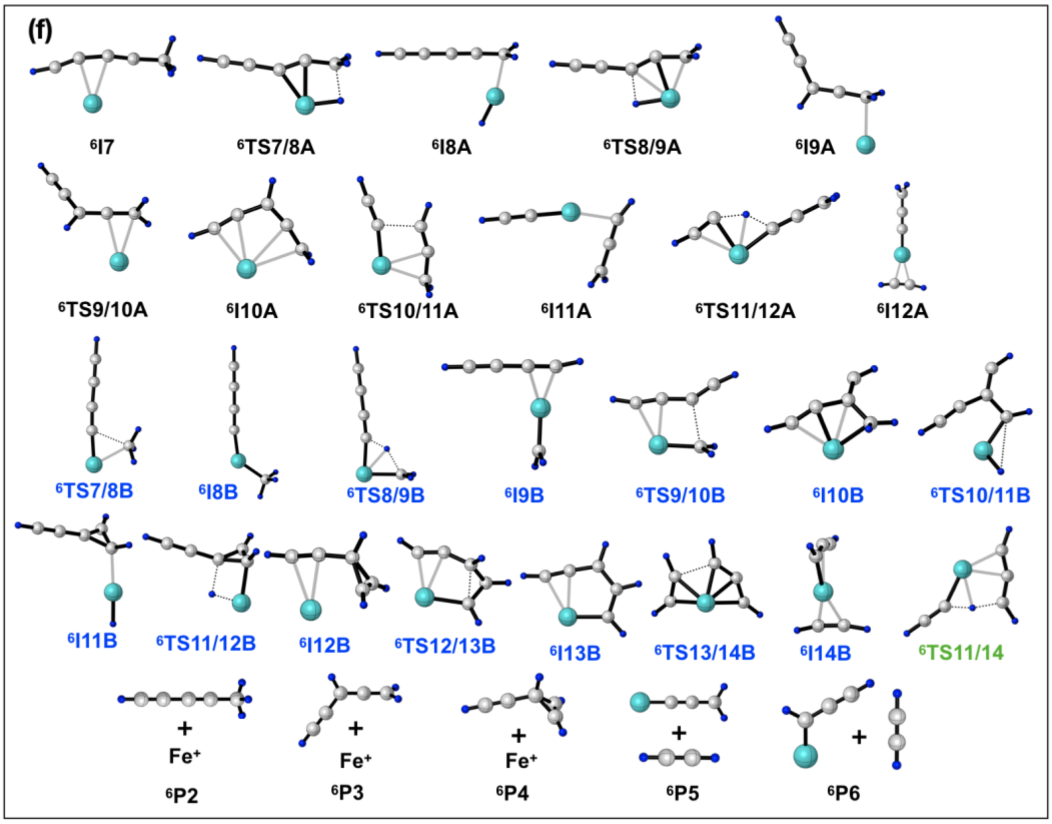
(f)
⁶I7
⁶TS7/8A
⁶I8A
⁶TS8/9A
⁶I9A
⁶TS9/10A
⁶I10A
⁶TS10/11A
⁶I11A
⁶TS11/12A
⁶I12A
⁶TS7/8B
⁶I8B
⁶TS8/9B
⁶I9B
⁶TS9/10B
⁶I10B
⁶TS10/11B
⁶I11B
⁶TS11/12B
⁶I12B
⁶TS12/13B
⁶I13B
⁶TS13/14B
⁶I14B
⁶TS11/14
Fe⁺
⁶P2
Fe⁺
⁶P3
Fe⁺
⁶P4
⁶P5
⁶P6

Figure S4. The selected active spaces considered in the NEVPT2(17e,15o)/QZ//CASSCF(17e,15o)/TZ calculations. Natural orbital partial occupation numbers are given.

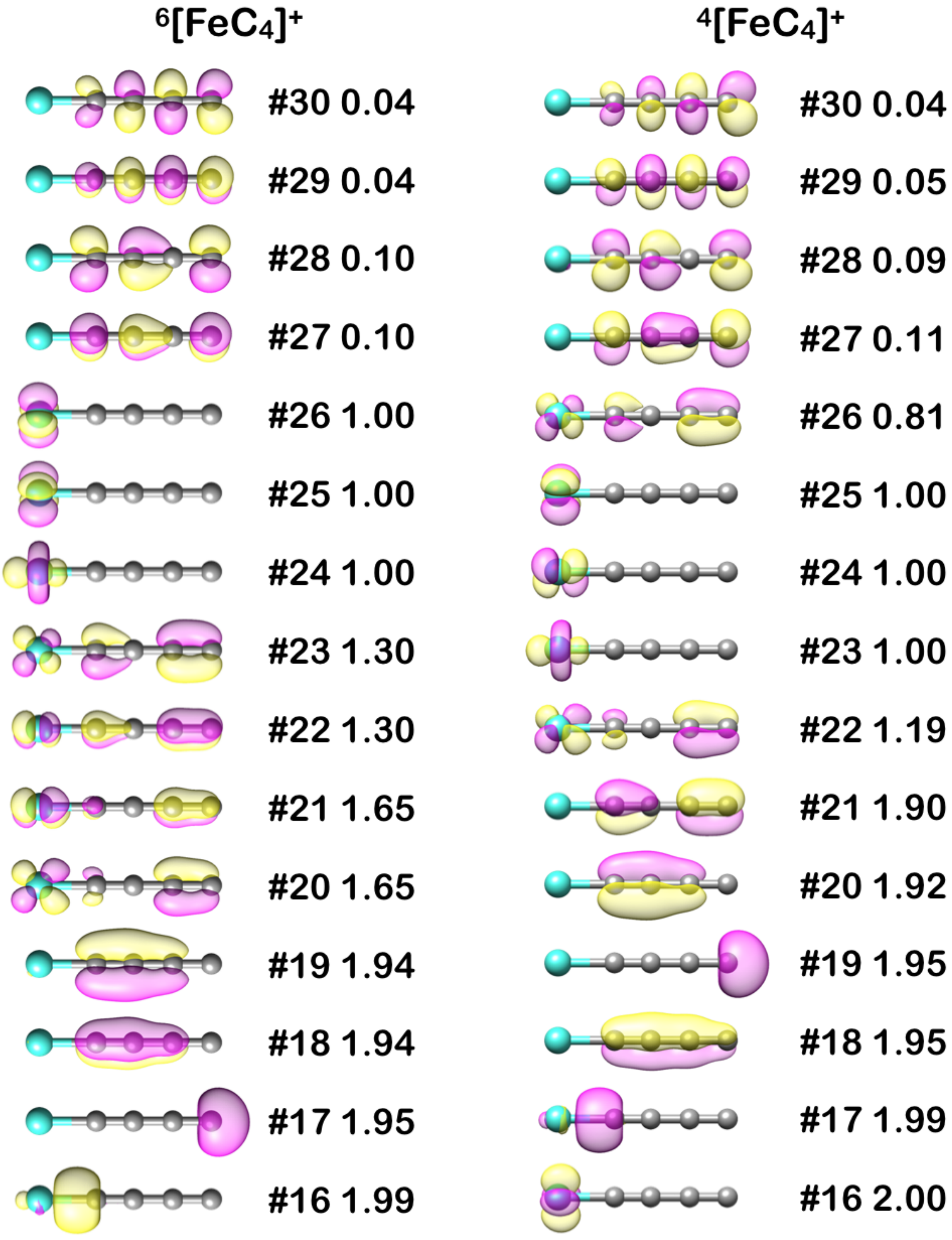

Figure S5. Schematic orbital diagrams represented by a frontier orbital analysis for the selected points in path D obtained by CASSCF(19e,17o) calculations. Natural orbital partial occupation numbers are also given.[a]

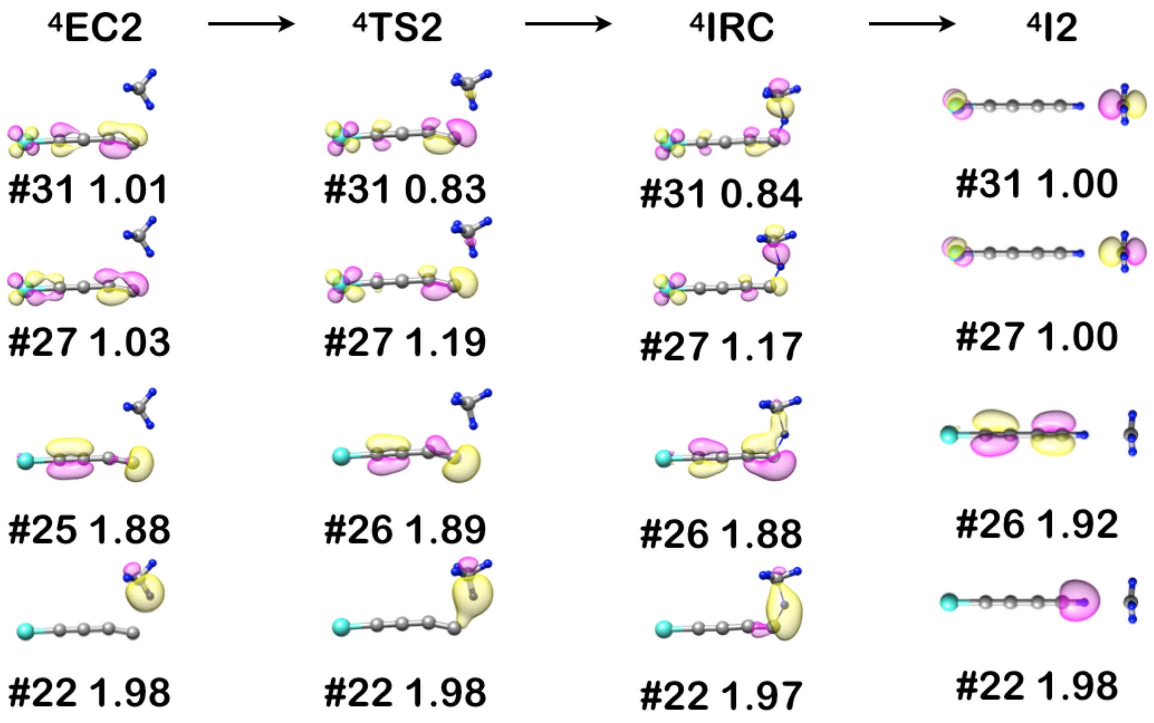

[a]Note, the orbitals of 27 and 31 are two individual singly-occupied orbitals but with significant overlaps to each other.

Figure S6. The evolution of the spin density of C_δ (red) and C_{CH_4} (black) along the sextet (a) and quartet (b) states reaction coordinates of the first C–H bond activation of methane.

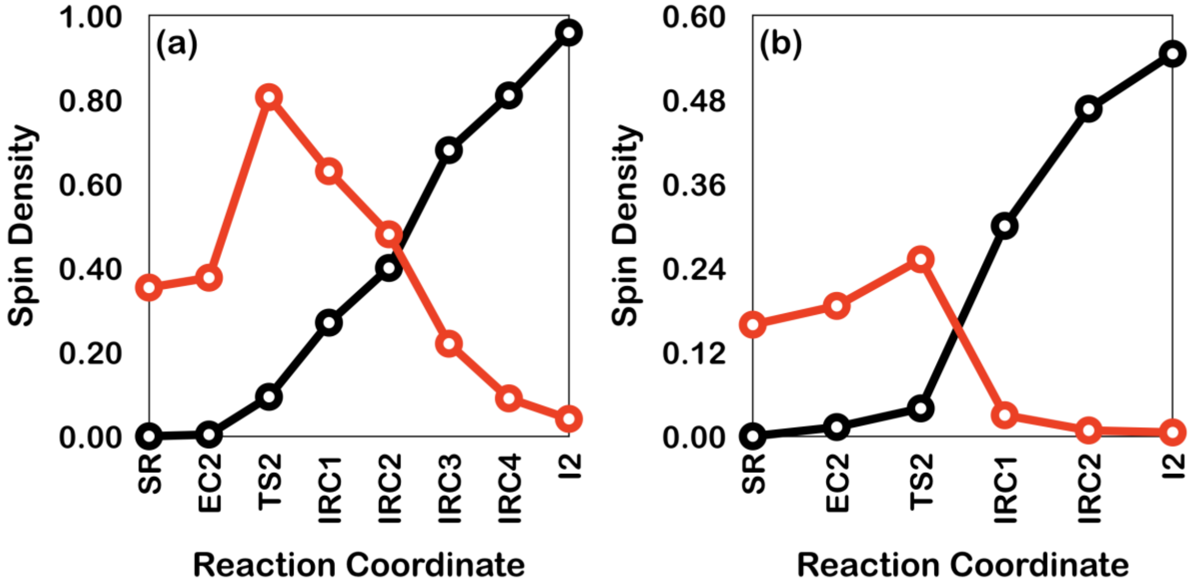

4. Tables

Table S1. Selected geometric parameters for ^{4,6}TS1 obtained by DFT methods combined with TZ basis set. Bond lengths are given in Angstrom.

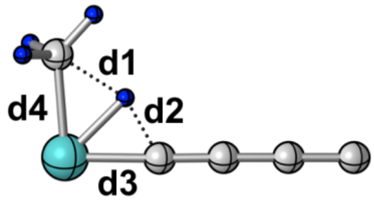

^{4,6}TS1

	^{6}TS1			
	d1	*d2*	*d3*	*d4*
ωB97	1.60	1.39	1.90	2.06
ωB97XD	1.58	1.40	1.91	2.08
MN15	1.60	1.39	1.89	2.07
MN15-L	1.61	1.44	1.85	2.08
BH&HLYP	1.53	1.40	1.98	2.12

	^{4}TS1			
	d1	*d2*	*d3*	*d4*
ωB97	1.50	1.40	1.75	1.95
ωB97XD	1.56	1.40	1.95	2.08
MN15	1.58	1.39	1.95	2.08
MN15-L	1.56	1.38	1.85	2.08
BH&HLYP	1.47	1.37	1.89	2.03

Table S2. Selected geometric parameters for ^{4,6}TS2 obtained by DFT methods combined with TZ basis set. Bond lengths are given in Angstrom and angles in degree.

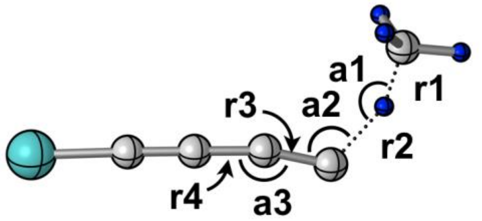

^{4,6}TS2

^{6}TS2							
	r1	*r2*	*r3*	*r4*	*a1*	*a2*	*a3*
ωB97	1.12	1.82	1.28	1.33	139	114	166
ωB97XD	1.14	1.68	1.29	1.30	156	114	166
MN15	1.18	1.49	1.28	1.32	155	120	168
MN15-L	1.27	1.36	1.29	1.32	160	123	167
BH&HLYP	1.10	1.92	1.26	1.33	144	114	165

^{4}TS2							
	r1	*r2*	*r3*	*r4*	*a1*	*a2*	*a3*
ωB97	1.12	1.84	1.29	1.32	135	108	165
ωB97XD	1.10	2.37	1.29	1.31	115	106	168
MN15	1.10	2.30	1.30	1.31	112	105	168
MN15-L	1.12	1.99	1.32	1.31	131	105	167
BH&HLYP	1.11	1.79	1.27	1.32	147	111	165

5. References

(1) Engeser, M.; Weiske, T.; Schröder, D.; Schwarz, H., Oxidative degradation of small cationic vanadium clusters by molecular oxygen: on the way from V_n^+ (n = 2 - 5) to VO_m^+ (m = 1, 2). *J. Phys. Chem. A* **2003,** *107*, 2855–2859.

(2) Schröder, D.; Schwarz, H.; Clemmer, D. E.; Chen, Y.; Armentrout, P. B.; Baranov, V. I.; Böhme, D. K., Activation of hydrogen and methane by thermalized FeO^+ in the gas phase as studied by multiple mass spectrometric techniques. *Int. J. Mass Spectrom.* **1997,** *161*, 175–191.

(3) Eller, K.; Schwarz, H., Organometallic chemistry in the gas phase. A comparative Fourier transform-ion cyclotron resonance/tandem mass spectrometry study. *Int. J. Mass Spectrom.* **1989,** *93*, 243–257.

(4) Frisch, M. J.; Trucks, G. W.; Schlegel, H. B.; Scuseria, G. E.; Robb, M. A.; Cheeseman, J. R.; Scalmani, G.; Barone, V.; Mennucci, B.; Petersson, G. A.; Nakatsuji, H.; Caricato, M.; Li, X.; Hratchian, H. P.; Izmaylov, A. F.; Bloino, J.; Zheng, G.; Sonnenberg, J. L.; Hada, M.; Ehara, M.; Toyota, K.; Fukuda, R.; Hasegawa, J.; Ishida, M.; Nakajima, T.; Honda, Y.; Kitao, O.; Nakai, H.; Vreven, T.; J. A. Montgomery, J.; Peralta, J. E.; Ogliaro, F.; Bearpark, M.; Heyd, J. J.; Brothers, E.; Kudin, K. N.; Staroverov, V. N.; Keith, T.; Kobayashi, R.; Normand, J.; Raghavachari, K.; Rendell, A.; Burant, J. C.; Iyengar, S. S.; Tomasi, J.; Cossi, M.; Rega, N.; Millam, J. M.; Klene, M.; Knox, J. E.; Cross, J. B.; Bakken, V.; Adamo, C.; Jaramillo, J.; Gomperts, R.; Stratmann, R. E.; Yazyev, O.; Austin, A. J.; Cammi, R.; Pomelli, C.; Ochterski, J. W.; Martin, R. L.; Morokuma, K.; Zakrzewski, V. G.; Voth, G. A.; Salvador, P.; Dannenberg, J. J.; Dapprich, S.; Daniels, A. D.; Farkas, O.; Foresman, J. B.; Ortiz, J. V.; Cioslowski, J.; Fox, D. J., Gaussian 09, Revision D.01, Gaussian, Inc., Wallingford CT, **2013.**

(5) Neese, F., Software update: the ORCA program system, version 4.0. *WIREs Comput. Mol. Sci.* **2018,** *8*, e1327.

(6) Ding, X.-L.; Li, Z.-Y.; Meng, J.-H.; Zhao, Y.-X.; He, S.-G., Density-functional global optimization of (La_2O_3)n clusters. *J. Chem. Phys.* **2012,** *137*, 214311.

(7) Zhu, W.; Li, G., Structures and properties of small iron-doped carbon clusters. *Int. J. Mass Spectrom.* **2009,** *281*, 63-71.

(8) Hegarty, D.; Robb, M. A., Application of unitary group methods to configuration interaction calculations. *Mol. Phys.* **1979,** *38*, 1795-1812.

(9) Weigend, F., Accurate Coulomb-fitting basis sets for H to Rn. *Phys. Chem. Chem. Phys.* **2006,** *8*, 1057-1065.

(10) Weigend, F.; Ahlrichs, R., Balanced basis sets of split valence, triple zeta valence and quadruple zeta valence quality for H to Rn: Design and assessment of accuracy. *Phys. Chem. Chem. Phys.* **2005,** *7*, 3297-3305.

(11) Angeli, C.; Cimiraglia, R.; Evangelisti, S.; Leininger, T.; Malrieu, J. P., Introduction of *n*-electron valence states for multireference perturbation theory. *J. Chem. Phys.* **2001,** *114*, 10252-10264.

(12) Chai, J. D.; Head-Gordon, M., Long-range corrected hybrid density functionals with damped atom-atom dispersion corrections. *Phys. Chem. Chem. Phys.* **2008,** *10*, 6615-6620.

(13) Chai, J.-D.; Head-Gordon, M., Systematic optimization of long-range corrected hybrid density functionals. *J. Chem. Phys.* **2008,** *128*, 084106.

(14) Truhlar, D. G.; Gordon, M. S., From force fields to dynamics: Classical and quantal paths. *Science* **1990,** *249*, 491-498.

(15) Gonzalez, C.; Schlegel, H. B., Reaction path following in mass-weighted internal coordinates. *J. Phys. Chem.* **1990,** *94*, 5523-5527.

(16) Fukui, K., The path of chemical reactions - the IRC approach. *Acc. Chem. Res.* **1981,** *14*, 363-368.

(17) Fukui, K., Formulation of the reaction coordinate. *J. Phys. Chem.* **1970,** *74*, 4161-4163.

(18) Becke, A. D., A new mixing of Hartree‐Fock and local density‐functional theories. *J. Chem. Phys.* **1993,** *98*, 1372-1377.
(19) Yu, H. S.; He, X.; Truhlar, D. G., MN15-L: A new local exchange-correlation functional for Kohn–Sham density functional theory with broad accuracy for atoms, molecules, and solids. *J. Chem. Theory Comput.* **2016,** *12*, 1280-1293.
(20) Yu, H. S.; He, X.; Li, S. L.; Truhlar, D. G., MN15: A Kohn–Sham global-hybrid exchange–correlation density functional with broad accuracy for multi-reference and single-reference systems and noncovalent interactions. *Chem. Sci.* **2016,** *7*, 5032-5051.
(21) Medvedev, M. G.; Bushmarinov, I. S.; Sun, J.; Perdew, J. P.; Lyssenko, K. A., Density functional theory is straying from the path toward the exact functional. *Science* **2017,** *355*, 49-52.

--

6. Coordinates

$^{6}[FeC_4]^+$

Fe	0.000012	0.000002	-1.762661
C	0.000012	0.000002	0.043415
C	0.000012	0.000002	1.323592
C	0.000012	0.000002	2.610154
C	0.000012	0.000002	3.920822

$^{4}[FeC_4]^+$

Fe	-3.725367	1.417683	-0.003878
C	-1.929177	1.161795	-0.000308
C	-0.676105	0.983331	0.002184
C	0.618433	0.798923	0.004759
C	1.907586	0.615612	0.007326

^{4}EC1

Fe	-1.152887	0.076986	0.111841
C	0.679815	0.042045	0.063209
C	1.937601	0.015588	0.018971
C	3.252168	-0.012175	-0.026254
C	4.546878	-0.041500	-0.075470
H	-3.119757	0.497333	0.947699
C	-3.358621	0.022386	-0.024032
H	-3.058324	-1.039019	-0.085695
H	-4.447603	0.011695	-0.059159
H	-3.048785	0.621098	-0.899386

^{6}EC1

Fe	-4.085773	1.172472	-1.465921
C	-3.032606	2.277156	-2.486693
C	-2.324187	3.023037	-3.167927
C	-1.543057	3.845595	-3.919346
C	-0.846556	4.578971	-4.589450
H	-6.022828	0.370453	-0.782170
C	-5.295917	-0.053676	-0.065067
H	-4.816301	0.704939	0.578268
H	-4.580045	-0.759309	-0.525618
H	-5.894411	-0.664848	0.609521

^{4}EC2

Fe	-2.375010	-0.181659	0.000077
C	-0.618253	0.245184	0.000302
C	0.604089	0.557795	0.000810
C	1.878255	0.881534	0.001350
C	3.034172	1.468368	0.002022
H	4.457517	-0.172915	-0.001325

C	4.152182	-1.229541	-0.000920
H	5.065572	-1.825344	0.001121
H	3.573232	-1.453678	0.895678
H	3.576206	-1.454869	-0.899115

^{6}EC2

Fe	1.714699	0.867878	-0.000279
C	0.130052	0.000251	0.000817
C	-0.992004	-0.614520	0.001610
C	-2.120029	-1.233600	0.002444
C	-3.258946	-1.882952	0.003349
H	-4.951732	0.213799	-0.003614
C	-4.390763	1.150877	-0.000794
H	-3.767765	1.210570	-0.895508
H	-3.772660	1.208150	0.897462
H	-5.094418	1.982950	-0.001574

^{4}TS1

Fe	3.272100	-1.762843	-0.058476
C	3.210144	-0.060326	0.358064
C	3.048781	1.163727	0.672046
C	2.957252	2.402370	1.037836
C	2.842530	3.644251	1.396846
H	2.374478	-0.585551	-0.638466
C	1.591428	-1.803119	-1.046344
H	1.976002	-2.535649	-1.773832
H	1.007719	-1.056426	-1.588414
H	0.966706	-2.250892	-0.262274

^{6}TS1

Fe	3.265681	-1.886558	0.088923
C	3.210521	-0.036331	0.497474
C	3.103465	1.214025	0.724121
C	3.008716	2.483994	0.964235
C	2.915421	3.759320	1.206659
H	2.245636	-0.610904	-0.325328
C	1.584795	-1.849586	-1.093333
H	2.018260	-2.260015	-2.014649
H	0.901819	-1.048286	-1.383457
H	0.992828	-2.610119	-0.567661

^{4}TS2

Fe	2.484526	0.347050	-0.000773
C	0.730766	-0.075154	-0.000373
C	-0.487154	-0.393297	-0.000089
C	-1.764991	-0.724143	0.000207
C	-2.883252	-1.366049	0.000572

H	-4.265461	-0.146656	0.000419
C	-4.343935	0.966052	0.000103
H	-3.879848	1.367801	-0.899421
H	-3.879570	1.368331	0.899248
H	-5.414637	1.173752	0.000208

^{6}TS2

Fe	2.637545	0.248567	-0.001089
C	0.810894	-0.130292	-0.000479
C	-1.700250	-0.697238	0.000381
C	-0.405968	-0.404413	-0.000064
C	-2.842414	-1.276530	0.000945
H	-4.251414	-0.124745	0.000688
C	-4.431941	0.979636	0.000065
H	-4.001756	1.416859	-0.899583
H	-4.001229	1.417964	0.898922
H	-5.517023	1.087882	0.000316

^{4}I2

Fe	3.060188	-1.292866	0.001366
C	1.294852	-0.861788	-0.000168
C	0.096856	-0.566748	0.000191
C	-1.226568	-0.241068	0.000443
C	-2.397807	0.047128	0.000638
H	-3.447853	0.305652	0.000835
C	-5.637925	0.847468	0.000190
H	-5.585640	1.377875	-0.942286
H	-5.582270	1.402773	0.928010
H	-5.973626	-0.181808	0.014502

^{6}I2

Fe	2.974912	-1.204093	0.012031
C	1.212983	-0.764972	0.006417
C	0.015007	-0.466299	0.003563
C	-1.306127	-0.136859	0.001109
C	-2.477328	0.155204	-0.000765
H	-3.528550	0.417298	-0.002416
C	-5.677026	0.951689	-0.003816
H	-5.647189	1.411901	-0.983495
H	-5.607135	1.574749	0.878999
H	-6.016409	-0.071979	0.092994
